# Prevalence and associated factors of active trachoma among children in Ethiopia: a systematic review and meta-analysis

**DOI:** 10.1186/s12879-019-4686-8

**Published:** 2019-12-21

**Authors:** Alemu Gebrie, Animut Alebel, Abriham Zegeye, Bekele Tesfaye, Fasil Wagnew

**Affiliations:** 1 Department of Biomedical Science, School of Medicine, Debre Markos University, P.O. Box 269, Debre Markos, Ethiopia; 2 Department of Nursing, College of Health Sciences, Debre Markos University, Debre Markos, Ethiopia

**Keywords:** Active trachoma, Associated factors, Children, Ethiopia

## Abstract

**Background:**

Trachoma is the commonest infectious cause of blindness. It is prevalent in areas where personal and community hygiene is poor, and it mainly affects deprived and marginalized communities most importantly in Ethiopia. Hence, the aim of this study was to determine the prevalence and associated factors of active trachoma among children in Ethiopia.

**Method:**

A systematic review and meta-analysis was employed to determine the prevalence of active trachoma and associated factors among children in Ethiopia. We searched databases, including PubMed, Google Scholar, Science Direct, EMBASE and Cochrane Library. To estimate the prevalence, studies reporting the prevalence of active trachoma and its associated factors were included. Data were extracted using a standardized data extraction format prepared in Microsoft excel and the analysis was done using STATA 14 statistical software. To assess heterogeneity, the Cochrane Q test statistics and *I*^*2*^ test were used. Since the included studies revealed considerable heterogeneity, a random effect meta- analysis model was used to estimate the pooled prevalence of active trachoma. Moreover, the association between factors and active trachoma were examined.

**Results:**

The result of 30 eligible studies showed that the overall prevalence of active trachoma among children in Ethiopia was 26.9% (95% CI: 22.7, 31.0%). In the subgroup analysis, while the highest prevalence was reported in SNNP (35.8%; 95% CI: 22.7, 48.8), the lowest prevalence was reported in Oromia region (20.2%; 95% CI: 12.2, 28.2). Absence of latrine: OR 6.0 (95% CI 2.0, 17.5), the unclean faces of children: OR 5.5 (95% CI 2.8, 10.9), and no reported use of soap for washing: OR 3.3 (95% CI 1.8, 6.0) have shown a positive association with active trachoma among children.

**Conclusion:**

From this review, it has been concluded that active trachoma among children is still a public health problem in different districts of Ethiopia. The prevalence of almost all studies are significantly higher than WHO target for elimination. Absence of latrine, unclean faces of children, no reported use of soap for washing are the important factors associated with active trachoma among children.

## Background

Trachoma is caused by the bacterium *Chlamydia trachomatis*, which is the commonest infectious cause of blindness in our globe [1–4]. The disease can be transmitted by the discharge from infected eyes of individuals and transferred by fingers, eye-seeking flies or by clothes to the eyes of non-infected ones. Trachoma is prevalent in areas where personal and community hygiene is poor, and it mainly affects deprived and marginalized classes of a community [5, 6]. As per the 2018 report of WHO weekly epidemiologic record, the number of people living in districts where active trachoma was a public health problem was 157.7 million, 88% of which in Africa and 50% (69,802,693) of which in Ethiopia [7].

Africa remains the most affected continent, and the one with the most intensive control efforts. In 2017, in the 22 countries of WHO’s Africa Region in which trachoma is known to be a public health problem, more than 226,000 people with trichiasis were given operations (95% of the global total operated on for trichiasis), and more than 79 million people were treated with antibiotics (95% of the global total given antibiotics for trachoma [https://www.who.int/news-room/fact-sheets/detail/trachoma]. Considering it as a public health problem, Ethiopia had signed the VISION 2020 Initiative in 2002 and developed its own 20 years strategic plan to eliminate trachoma as a public health problem [8].

Although they vary among settings depending on economical, personal as well as environmental reasons, several factors are associated with an increased prevalence of trachoma. These include absence of hygiene and sanitation facilities and services, scarcity of water, living in trachoma endemic areas, overcrowded, living environment and poverty [9]. Studies conducted in Africa showed that children with ocular and nasal discharges were more likely to suffer from trachoma [10, 11]. Either clean faces with eye seeking flies or unclean faces, time to fetch water, garbage in the compound, overcrowding, children with age 3–5 years, less frequent face washing, presence of cattle in the household, open defecation, high fly density in the household are some of the factors associated with active trachoma in the studies [12–15].

The World Health Organization (WHO) in collaboration with other national health services and non-governmental organizations (NGOs) started implementation of a program to eliminate trachoma as a public health problem (Global Elimination of Trachoma by 2020) [11]. The strategy of the program, termed as SAFE, comprises these control measures: Surgery for trichiasis (extremely painful condition), Antibiotics for infectious trachoma, Facial cleanliness to reduce transmission and Environmental improvement like control of disease transmitting eye seeker flies and the access to clean water [11].

In different regions of Ethiopia, several independent studies were conducted in children to assess the prevalence and associated factors of trachoma, but there was a great variation and inconsistency of the findings among the studies. To the best of our knowledge, the reasons for the variation of the prevalence and associated factors of trachoma in children in the studies have not yet been assessed. Hence, the aim of this systematic review and meta-analysis was to review and determine the prevalence and associated factors of active trachoma among children in Ethiopia. The results of the present study will help policy makers and other concerned bodies to plan and campaign for the improvement of programs aimed at trachoma prevention and eradication in the country. The study will also help researchers to carry out related studies with more interventional study designs. The review question is: What is the best available evidence on the prevalence and associated factors of active trachoma among children in Ethiopia?

## Methods

### Study design and literature searching strategies

This review was conducted to compile the most contemporary evidences using published articles as well as grey literature on the prevalence and associated factors of active trachoma among children in Ethiopia. For reporting, we followed the protocol of the Preferred Reporting Items for Systematic Reviews and Meta-Analyses (PRISMA) guideline [16]. Two approaches were followed to search potentially relevant studies. The electronic database search (PubMed, Google scholar, Science Direct, EMBASE, Cochrane library) and Google Scholar), and the manual search of references lists of previous prevalence studies to retrieve more related articles. “Associated factors”, “Trachoma”, “Active trachoma”, “Children” and “Ethiopia” (see Additional file 1) were the key terms used for searching the articles in the reputable databases used both in separation and in combination using the Boolean operator like “OR” or “AND”. The articles were searched from 15th September, 2017 to 31st December, 2018 and all the articles accessed until 31st December, 2018 were included in the study.

### Eligibility criteria

#### Inclusion criteria

The two investigators (AG and AA) independently and carefully reviewed the contents of each retrieved articles. Those literature fulfilling the following criteria were finally included in the study.

##### Population

Studies done among children (age < 15) were considered.

##### Study area

Only those studies that were carried out in Ethiopia were included.

##### Study design

Original articles that contain data reporting the prevalence and associated factors of active trachoma among children in Ethiopia were eligible.

##### Language

Literature which have been published in English language were considered.

##### Publication condition

Articles that met the criteria were considered irrespective of their publication status i.e. published, unpublished or grey literature).

#### Exclusion criteria

Three independent reviewers carried out the data extraction blindly after assessment of the abstracts and the full texts of the literature. Articles with problems in methods were excluded by the two independent researchers after reading the full text as well as abstracts. Because of incomplete data, full text inaccessible articles were not included in the review.

#### Data extraction

Using a pre-tested data extraction format, the necessary data were extracted by the investigators. The data extracted from the studies included: first author, region in the country where the study was conducted, the specific study area, study design, year of publication of the study, the sample size, response rate of the study and active trachoma prevalence. Any disagreements between the two authors on extraction of the data were settled through discussion and consensus. In addition, the variation was also resolved by involving third reviewer.

### Operational definitions of outcomes

The prevalence of active trachoma (defined here as trachomatous inflammation—follicular or trachomatous inflammation—intense) among children was the main outcome of this review. The second outcome of the study was to assess the factors that are associated with active trachoma in children. In the included studies, we took the prevalence of trachoma in children that is adjusted by weighting the proportion of each 1-year age band observed to have TF by the proportion of the local 1–9-year-old population expected to have that age, according to the most recent census. The association between active trachoma and factors were examined through odds ratio. The odds ratio was calculated from the report of two by two Tables OR = ad/bc.

### Quality assessment

To evaluate the quality of the studies, the authors used the Newcastle-Ottawa quality assessment tool Scale adapted for cross-sectional studies [17].. The tool has three indicators. The first section is graded out of five stars and assesses the quality of the methodology of a study. The second part of the tool is graded out of three stars and assesses the comparability of the studies. The last part of the tool is graded from two stars and measures the quality of the original articles with respect to their statistical analyses. Using the tool as a protocol two independent authors evaluated the quality of the original articles. Those studies with medium (fulfilling 50% of quality assessment criteria) and high quality (≥6 out of 10 scales) were included for analysis. By taking the mean score of the two researchers, disagreements of their assessment results were resolved.

### Statistical analysis

The necessary data were extracted from the studies using Microsoft Excel 2016 format and then analyzed by using STATA version 14.0 (STATA Corporation, College Station Texas) software respectively. The primary articles were summarized by using tables and the forest plot. The researchers calculated the standard error of prevalence of active trachoma for each original article using the binomial distribution formula. We checked heterogeneity among the reported prevalence of the studies by using Cochrane Q statistics and I^2^ test [18]. Heterogeneity is quantified as high (considerable), moderate, low with ranges of 75% or more, 50–75% and 25% or less for I^2^, respectively [19]. A random effects model was considered to estimate the Der Simonian and Laird’s pooled effect since the test statistics showed there was a considerable heterogeneity among the studies (I^2^ = 97.5%, *p* = 0.000). Univariate meta-regression analysis was undertaken by taking the publication year and the sample size to identify the possible source (s) of heterogeneity and the authors found publication year to be statistically significant (*p* = 0.002). Potential publication bias was also assessed subjectively by funnel plot and objectively using Egger’s weighted correlation and Begg’s regression intercept tests at 5% significance level [20, 21]. In Egger’s and Begg’s tests *p*-value < 0.05 indicates the presence of publication bias while *p*-value > 0.05 indicates that there is no publication bias. A funnel plot which is plotted by effect estimate (prevalence in this study case) versus standard error of effect estimate, is another subjective assessment of publication bias. Each dot represents a single study and symmetric dots of inverted funnel shape shows the absence of publication bias. If publication bias is noticed in the Random-effects model, the estimate is determined by using Duval and Tweedie’s Trim and Fill analysis. In addition, subgroup analysis was done using region of studies.in order to reduce the random heterogeneity between the estimates of the primary studies.

## Results

### Searching results

**Fig. 1:**
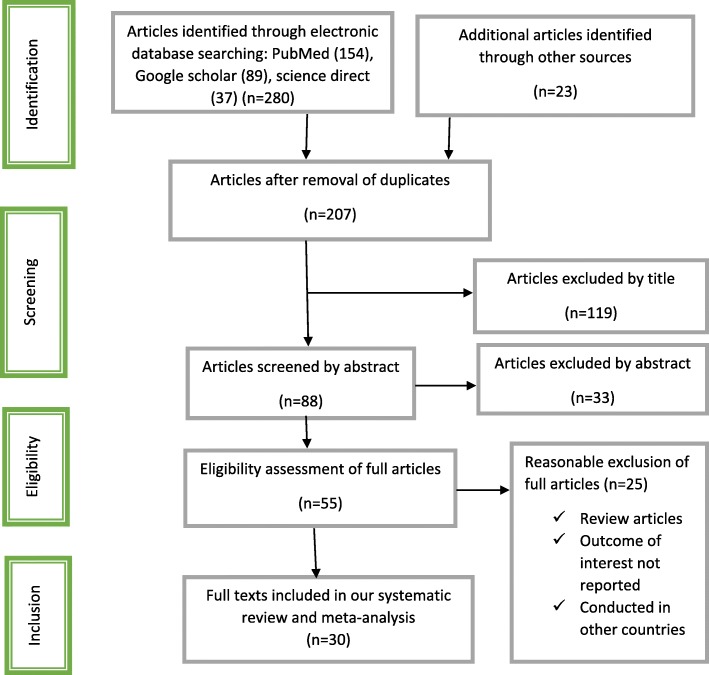
Flow chart diagram describing selection of studies for the systematic review and meta-analysis of prevalence and associated factors of active trachoma among children in Ethiopia, 2018 (identified, screened, eligible and included studies). Articles may have been excluded for more than one reason

The authors retrieved a total of 303 retrievals by search engines of databases. Because of duplications in the retrievals, 96 of them were excluded from inclusion. After evaluating the titles and abstracts, the rest 152 records, 207 retrievals were excluded because they were irrelevant for this meta-analysis regarding outcome of interest. Then, 55 full text articles were assessed for eligibility based on the consideration criteria. Finally, 30 studies were purported to be eligible for this systematic review and meta-analysis (Fig. 1).

From a total of 55 full text articles accessed, we rejected twenty of them because they were review studies, and /or conducted in other countries like: USA [22], China [23], India [24, 25], Nepal [26], Nigeria [27–29], Kenya [6, 30–32], South Sudan [33, 34], Tanzania [35, 36], Senegal [37], Malawi [38–40], Mali [41], and Gambia [42]. Moreover, five full text articles [43–47] that have been conducted from various regions across Ethiopia were not included since their outcome estimates were different from prevalence of active trachoma in children which is the outcome of interest of this study, and they have been done in the adult population that is not the population of interest of the current study.

### Description of original studies

The characteristics of the 30 primary studies included in this review has been described in Table 1. All of the studies were cross sectional study designs carried out in different parts of Ethiopia having sample size in a range of 267 in Dawro zone, Oromia region [67] to 62,869 in Amhara region [56]. These studies were conducted from 2005 to 2018. In the present meta-analysis, a total of 228,420 children were included to estimate the overall prevalence of active trachoma.

**Table 1:** Descriptive summary of 30 studies reporting the prevalence and associated factors of active trachoma among children in. Ethiopia included in the systematic review and meta-analysis, 2018

Region	Area	Author	Publication year	Sample size	Response rate (%)	Quality score (10 pts)	Prevalence (95% CI)
Amhara	Dembia District	Ferede et al [48]	2017	681	98	7	18.2 (15.3,21.3)
	Baso Liben	Ketema et al. [49]	2012	792	100	7	24.1 (21.1,27.1)
	Gazegibela	Anteneh and Getu [50]	2016	601	100	8	52.4 (48.4,56.4)
	Wollo	Tadesse et al. [51]	2017	1358	100	7	21.6 (19.4,23.8)
	Gonji Kolella	Nigusie et al. [52]	2015	618	100	7	23.1 (19.8,26.5)
	Maksegnit	Shiferaw and Moges [53]	2013	420	99.8	7	23.81 (19.7,27.9)
	ankober	Golovaty et al. [54]	2009	510	NR	8	53.9 (49.6,58.3)
	Dangla	Gedefaw et al. [55]	2013	409	100	6	12.0 (8.8,15.1)
	Belesa	Alemayehu	2005	1244	100	7	42.4 (39.6,45.1)
	Regionwide	William Oswald et al. [56]	2017	62,869	91	8	29.0 (28.7,29.4)
	Regionwide	Emerson et al. [57]	2008	5485	NR	8	32.7 (31.5,33.2)
Afar	Regionwide	Negash et al. [58]	2018	6399	NR	6	9.6 (8.8,10.3)
BG	Regionwide	Adamu et al. [59]	2016	7417	NR	6	8.3 (7.7,8.9)
Dredawa	Dera Woreda	Metadel et al. [60]	2015	671	96.5	8	15.7 (12.9,18.4)
Ethiopia	Nationwide	Birhane et al. [8]	2006	9289	NR	6	40.1 (39.2,41.1)
Gambella	Regionwide	Abashawl et al. [61]	2016	3238	NR	6	17.2 (16.9,18.5)
Harari	Harari region	Assefa et al. [62]	2017	1722	93.8	6	1.3 (0.8,1.8)
Oromia	Mojo and Lume	Kassahun Yalew et al. [63]	2012	431	100	5	22.5 (18.6,26.5)
	Kersa District	Meseret et al. [64]	2013	305	NR	5	25.3 (20.4,30.1)
	butajira	Mehari [65]	2014	735	NR	6	7.6 (5.7,9.5)
	Regionwide	Bero et al. [66]	2016	41,642	NR	6	23.4(23.0,23.8)
	Dawro Zone	Admasu W et al. [67]	2015	267	100	6	22.9 (17.8,27.9)
	Regionwide	Adera et al. [68]	2016	41,155	NR	6	28.3 (27.9, 48.8)
SNNP	Zala district	Mengstu et al. [69]	2016	611	98.87	7	36.7 (32.8,40.5)
	Goro district	Mohammed and Abebe [70]	2005	826	NR	5	34.9 (31.6,38.1)
	Gurage	Admassu et al. [12]	2013	768	97	8	22.8 (19.8,25.8)
	Gurage	Alemayehu et al. [71]	2005	2788	95	7	56.5 (54.7,58.3)
Somali	Regionwide	Duale et al. [72]	2018	23,620	NR	6	15.0 (14.5, 15.4)
Tigray	Regionwide	Sherief et al. [73]	2016	10,023	NR	6	26.7 (25.8, 27.5)
	Regionwide	Mesfin et al. [74]	2006	1526	NR	7	59.2 (56.8,61.7)

The 30 studies were conducted in different regions of the country: most of the studies were conducted in Amhara region: Amhara [48–57, 60], Tigray [73, 74], Harari [62], Oromia [63–67], Benishangul Gumuz [59], Afar [58], Somali [72], Gambella [61], Southern Nations, Nationalities and peoples’ region (SNNPR) [12, 68–71] and one nationwide study [8]. Whereas the highest prevalence of active trachoma in children (59.2%) was reported in Tigray region by one-region wide study [74], a study conducted in Harari [62] reported the lowest prevalence (1.28%) of the disease. Moreover, the original studies included in the meta-analysis and reporting response rate have had a response rate not less than 91% indicating that the studies have favorable response rate.

As far as the publication status of the studies is concerned: only one (Mesfin A: Assessing the prevalence of active trachoma among young children in relation to the implementation of safe strategy in EBINAT and EAST BELESA WOREDA, north West Ethiopia. unpublished) of the 30 studies was unpublished article (from Addis Ababa University digital repository library) and 29 of the studies included in the meta-analysis were searched by reproducible search from databases like PubMed. Independent evaluators re-assessed all the articles before any analysis and the studies were fit in terms of their quality (quality score ranged from 5 to 8 out of 10 points).

### Meta-analysis

**Fig. 2:**
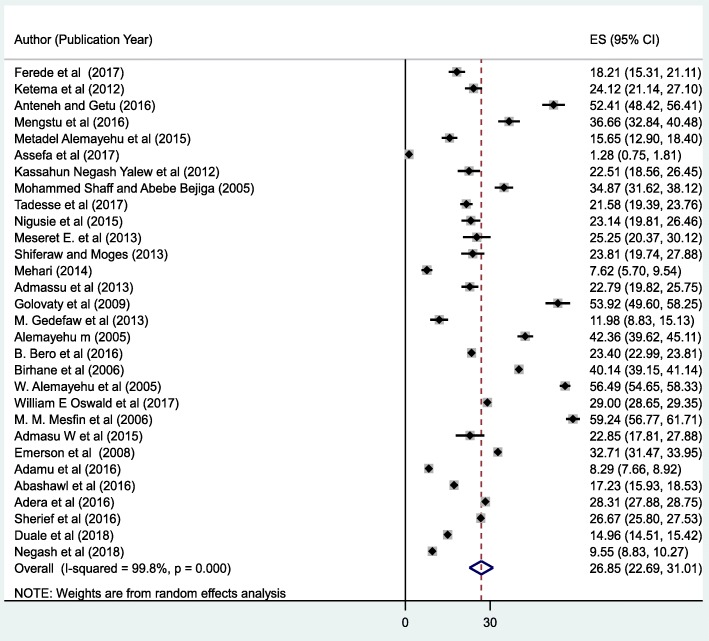
Forest plot of the pooled prevalence of active trachoma among children in, 2018

**Fig. 3:**
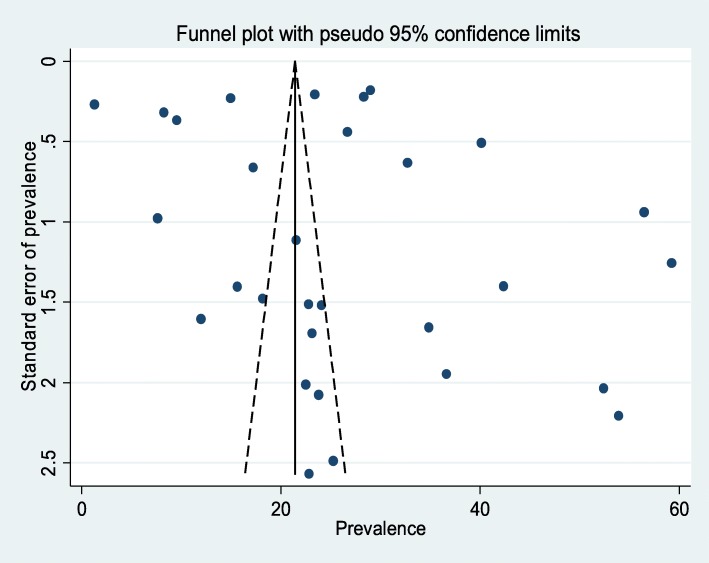
The funnel plot of the meta-analysis containing 30 studies

In this meta-analysis, the forest plot result of 30 included studies showed that the overall pooled prevalence of active trachoma among children in Ethiopia was 26.9% (95% CI: 22.7, 31, 0%) (Fig. 2). Nevertheless, considerable heterogeneity was seen among the studies and objectively detected by I^2^ statistic (I^2^ = 99.8, *p* value < 0.000). Therefore, a random effect model was used to estimate the overall pooled prevalence of active trachoma among children in Ethiopia. To explore the possible sources of heterogeneity, different factors potentially associated with the heterogeneity, such as publication year and sample size were checked by using univariate meta-regression models and publication year was statistically significant(*p* = 0.001) (Table 2). Begg’s and Egger’s tests revealed the absence of statistically significant publication bias (*p* = 0.80) and (*p* = 0.42) respectively. The funnel plot has also shown that the dots, representing the studies, were symmetrical that indicated the absence of publication bias (Fig. 3).

**Table 2:** Related factors with heterogeneity of active trachoma prevalence among children in Ethiopia in the current meta-analysis (based on univariate meta-regression)

Variables	Coefficient	P-value
Publication year	−3.582699	0.002
Sample size	0.000554	0.067

### Subgroup analysis

**Fig. 4:**
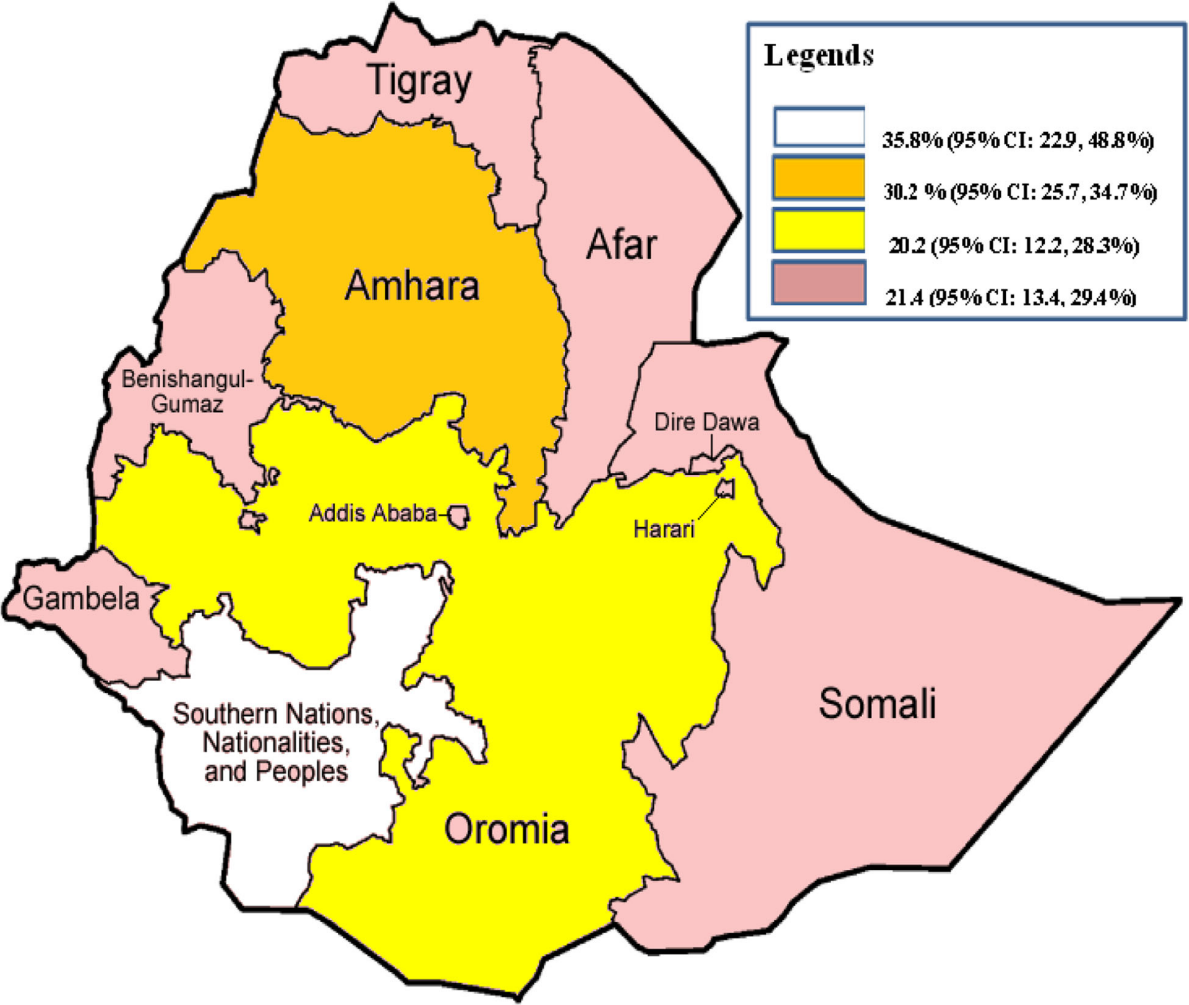
The map showing the distribution of active trachoma in regions of Ethiopia

In addition, in this meta-analysis, we performed subgroup analysis to explore sources of heterogeneity based on the region where the studies were conducted and publication years of the studies. Accordingly, the highest prevalence was reported in SNNP region with a prevalence of 35.8% (95% CI: 22.9, 48.8) followed by Amhara region where most of the studies have been conducted, 30.2% (95% CI: 25.7, 34.7) and Others, 21.4% (95% CI: 13.4, 29.4) (Table 3, Fig. 4). Concerning year of publication, the prevalence of active trachoma among children was significantly higher in studies which had been conducted before 2012 (32.7%; 95% CI: 24.8, 40.6) as compared to those studies which have been carried out since 2012 (21.7, 95% CI: 16.6, 26.8) (Table 3).

**Table 3:** Subgroup analysis of the prevalence active trachoma among children in Ethiopia, 2018 (*n* = 30)

Variables	Characteristics	Number of studies	Prevalence with 95%
Region	Oromia	5	20.2 (12.2, 28.3)
	Amhara	11	30.2 (25.7, 34.7)
	SNNP	5	35.8 (22.7, 48.8)
	Others	9	21.4 (13.4, 29.4)
Study year	< 2012	14	32.7 (24.8, 40.6)
	> 2012	16	21.7 (16.6, 26.8)

### Factors associated with active trachoma among children

**Fig. 5:**
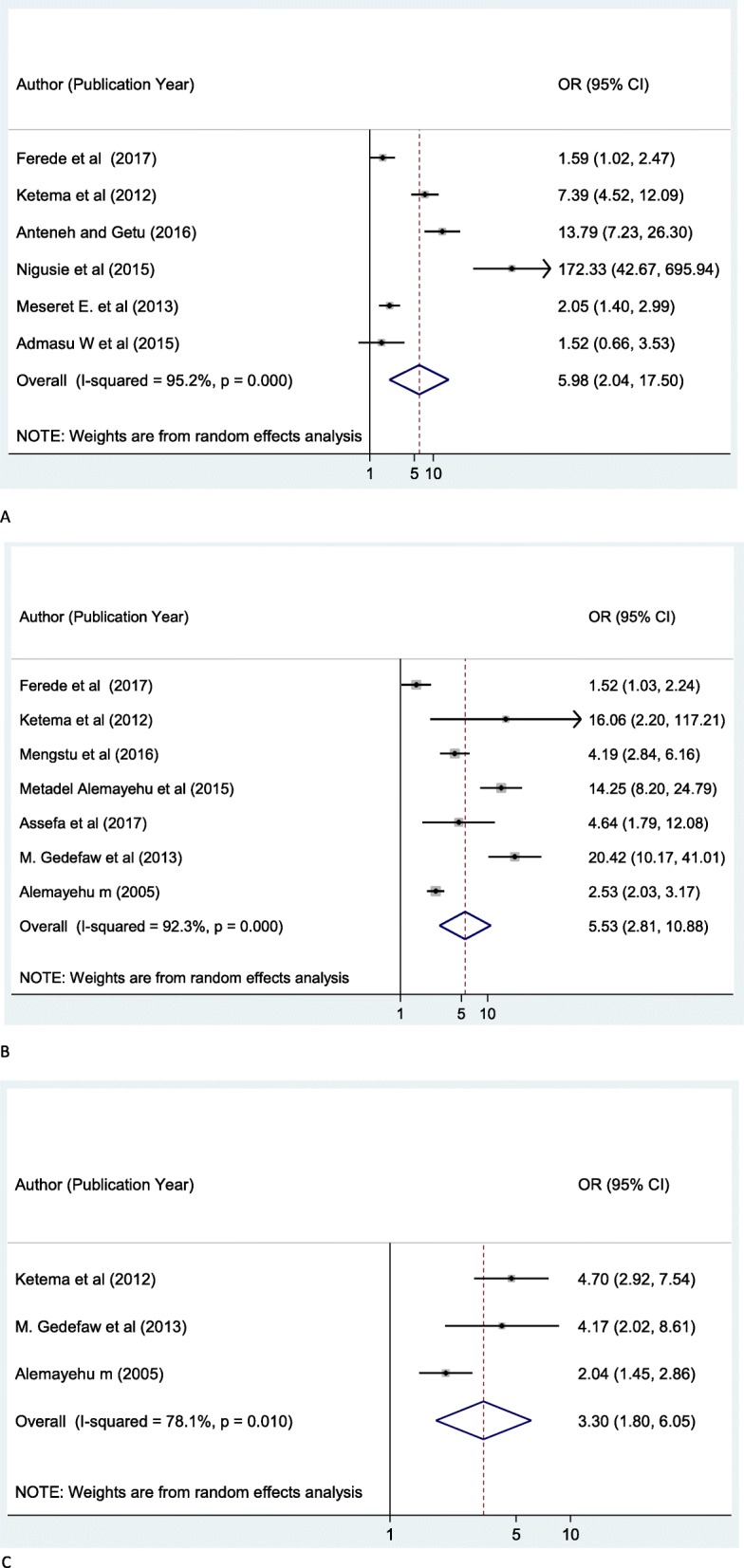
Forest plot depicting pooled odds ratio (log scale) of the associations between active trachoma among children in Ethiopia and its purported associated risk factors **a**: Absence of latrine, **b**: Face uncleanliness of children, **c**: No usage of soap)

Using a total of eleven pre and post intervention surveys [48–50, 52, 55, 60, 62, 64, 67, 69] (Mesfin A: Assessing the prevalence of active trachoma among young children in relation to the implementation of safe strategy in EBINAT and EAST BELESA WOREDA, north West Ethiopia. unpublished) with data that can be analyzed, we meta-analyzed the associations of the absence of latrine, the facial cleanliness of children, and the reported use of soap with active trachoma among children in Ethiopia (Fig. 5). The researchers also performed sensitivity analysis in the factors analyses and found no significant outlier from the studies.

In the analysis of the six pre and post intervention studies, we have found that the absence of latrine was significantly associated with active trachoma among children, OR: 5.9 (95% CI 2.0–17.5 Children who are living within the house of no latrine were 5.9 times more likely to have active trachoma as compared to those children who have access to functional latrine (Fig. 5a). The pooled result of seven studies also revealed that the facial cleanliness of children was strongly associated with active trachoma among children (Fig. 5b). Those children whose face has not been clean were 5.5 times more likely to have active trachoma as compared to their counterparts, odds ratio 5.5 (95% CI 2.8,10.9).

In addition, results from the meta-analyses of three pre-intervention studies (Fig. 5c) have shown that no reported use of soap was a significant factor for active trachoma among children in Ethiopia. Children who failed to use soap for washing were about 3.3 times more likely to have active trachoma as compared to those children who reported using soap while washing their body, odds ratio 3.3 (95% CI 1.8, 6.0).

Although they are not meta-analyzable, poor waste disposal, water inaccessibility, inadequate knowledge of family head about trachoma, keeping cattle near house, low monthly income and low altitude were also associated with active trachoma before and after interventions. Density of flies, illiteracy and using wood and animal dung for cooking were the factors associated with active trachoma in pre-intervention surveys. On the other hand, ocular discharge, family size, younger age and female sex were the factors associated with active trachoma in our systematic review of post intervention surveys.

## Discussion

Trachoma remains a leading cause of preventable blindness in underprivileged communities in different parts of Africa most importantly in Ethiopia. The problem is worse if it is not detected and intervened early in children. This study aims to estimate the pooled prevalence of active trachoma and identify factors that are associated with it among children in Ethiopia. Using 30 relevant studies, this meta-analysis estimated that the prevalence of active trachoma among children in Ethiopia was 26.9%, which is higher than WHO target for elimination (< 5%) in an evaluation unit of a country. In spite of the fact that SAFE strategy (Surgery for trichiasis, Antibiotics for active disease, Facial cleanliness, and Environmental improvement) has been implemented in Ethiopia to reduce the spread of the disease, active trachoma is still a public health burden [8].

The possible plausible reason for the situation could be owing to scarcity of clean water supply in many areas of the community. Moreover, about 48% of the population lack functional latrine access and virtually all the community experience open field animal waste product disposal [75]. Another possible reason could also be that health education and health promotion on primary health care specifically on eye, personal as well as environmental hygiene and sanitation in the communities may not be satisfactory.

The subgroup analysis of this study also showed that the prevalence of active trachoma among children significantly varies across regions of Ethiopia. The prevalence was higher in children living in SNNP (37.7%) and Amhara (30.2%) regions as compared to other regions of the country. This pinpoints that despite SAFE strategy implementation in Amhara region, the disease is still in need of campaign and SNNP region is yet another part of Ethiopia where the SAFE strategy is to be feasibly implemented. With regard to study year, the prevalence of active trachoma was significantly lower in those studies conducted since 2012 as compared to those studied before 2012. We hypothesize that this difference is due to implementation of the SAFE strategy in Amhara region. Other potential explanations include the (1) more heavily-endemic areas were surveyed first, because that was where the greatest need was perceived, and (2) different survey methodologies and methods of analysis. Nearly all surveys undertaken in Ethiopia since 2012 have used highly quality-controlled and quality assured methodologies [76].

We have also systematically reviewed and meta-analyzed the factors associated with active trachoma among children that have been addressed by the included studies. In the studies that examined the associated factors, presence of latrine, facial cleanliness and reported use of soap were meta-analyzable, and significantly associated with prevalence of active trachoma among children. Children that are from families who did not use latrine were 6.0 times more likely to be infected by active trachoma than those children with families who had access to functional latrine. This finding is in trajectory with a study carried out by WHO [77]. This could be reasoned out that inadequate disposal of waste materials is a factor for the presence of high number of eyes seeking flies that leads to a high chance of transmission of active trachoma.

Facial cleanliness of the children was also significantly associated with the development of active trachoma. Children with unclean faces were 5.5 more likely to have trachoma than those children whose faces were clean. The finding is concordant to a study identifying the presence of nasal and ocular discharge as factors for the presence of flies on eyes and active trachoma in Gambia and Tanzania [10]. The possible explanation is because of the fact that unclean faces attract eye-seeking flies which are potential mechanical vectors of *Chlamydia trachomatis* infection [78]. Nasal and ocular discharges may both result from the inflammation of active trachoma and cause the face to be classified as unclean [22, 79, 80].

Moreover, the result of this study (review of pre-intervention surveys) showed that face washing using soap has a protective association against active trachoma among children. Children who were not using soap while washing their face were 3.3 times more likely to suffer from trachoma than those children who were using soap during face washing.

## Conclusions

From this review, it has been concluded that active trachoma among children is still a public health problem in different districts of Ethiopia although Ethiopia signed the VISION 2020 Initiative in 2002 and developed its own 20 years’ strategic plan to eliminate trachoma as a public health problem. The prevalence of almost all studies are significantly higher than WHO target for elimination. Absence of latrine, facial uncleanliness and no reported use of soap for washing are factors associated with active trachoma among children. Therefore, based on our findings, we recommend that the government and other concerned bodies shall undertake successful SAFE strategy implementation integrated with health education and health promotion to prevent and eliminate trachoma as a public health problem from the country.

## Supplementary information

**Additional file 1.** Searching approach for PubMed

## Data Availability

All relevant data are within the paper and its Supporting Information files.

## Abbreviations

BG: Benshangul Gumuz
PRISMA: Preferred Reporting Items of Systematic Reviews and Meta-Analysis
SNNPE: Southern Nations, Nationalities and peoples of Ethiopia
USA: United States of America
WHO: World Health Organization
