# Supplementary material for: Prevalence and associated factors of active trachoma among children in Ethiopia: a systematic review and meta-analysis
Source: BMC Infect Dis. 2019 Dec 21;19:1073. doi: 10.1186/s12879-019-4686-8 (PMC6925509; doi:10.1186/s12879-019-4686-8)
Supplement: Supplementary file 1 — Additional file 1. Searching approach for PubMed [file 12879_2019_4686_MOESM1_ESM.docx]

| **Additional file**  **Searching approach for PubMed** | |
| --- | --- |
| **Study Subject** | (Preschool OR Children OR ‘School children’ OR ‘School aged’ OR Childhood OR Schooler OR Preadolescent OR kindergarten) |
| **Outcome of interest** | (Trachoma OR Active trachoma OR ‘TF’ OR ‘TI |
| **Study type** | (Epidemiology OR Prevalence OR trend) |
| **Study design** | (‘Cross-sectional’ OR Observational OR Longitudinal OR Survey or census NOT (Review OR ‘Case report’ OR ‘Case series’)) |
| **Location** | (Ethiopia OR Tigray OR Afar OR Amhara OR Oromia OR ‘Ethiopian Somali’ OR ‘Benshangul/Gumz’ OR SNNPs OR Gambela OR Harari OR ‘Addis Ababa’ OR ‘Dire Dawa’) |
